# Exploring the psychosocial impact of work-life balance among nurse educators in a Gauteng Nursing Education Institution

**DOI:** 10.3389/fpubh.2026.1790115

**Published:** 2026-04-21

**Authors:** Letta Mathebula, Rirhandzu Friddah Mathevula, Tshiamo Nerville Ramalepa

**Affiliations:** Nursing Science Department, Sefako Makgatho Health Sciences University, Pretoria, South Africa

**Keywords:** Gauteng, nurse educators, Nursing Education Institution, psychosocial, work-life balance

## Abstract

**Background:**

Work-life balance (WLB) is a critical determinant of psychosocial well-being among nurse educators, particularly in high-pressure Nursing Education Institutions (NEIs). In South Africa, nurse educators face mounting demands from teaching, clinical supervision, and administrative duties, often resulting in emotional exhaustion and stress.

**Aim:**

This study explored the psychosocial impact of work-life balance among nurse educators in Gauteng, with a focus on developing support strategies.

**Setting:**

Selected Nursing Education Institution in Gauteng Province, South Africa.

**Methods:**

A qualitative, exploratory, and descriptive research design was employed, involving semi-structured interviews with purposively selected non-probability nurse educators. Data was analysed thematically to identify key psychosocial challenges related to work-life balance.

**Results:**

The analysis revealed two primary themes: the first pertained to the negative consequences of stress and burnout, including adverse effects on physical and mental health, as well as emotional exhaustion stemming from work-related frustrations. The second theme highlights the inadequacies of support systems, including limited access to counseling services, insufficient support for academic pursuits, and social challenges.

**Conclusion:**

The study highlighted the urgent need for institutional reforms that promote psychosocial resilience among nurse educators. Strategies such as flexible scheduling, peer support networks, and wellness programmes may enhance work-life balance and improve educational outcomes. These insights are vital for informing policy and practice within South African nursing education.

## Introduction

Work-life balance is crucial for psychosocial well-being, especially in high-stress professions like nursing education. Nurse educators handle the demands of delivering quality academic programmes while managing clinical and administrative duties, which can lead to chronic stress, emotional exhaustion, and burnout (1, 2). These challenges are especially pronounced in South African NEIs, where resource constraints and systemic pressures exacerbate role conflict and diminish WLB. In KwaZulu-Natal, more than 60% of nurse educators reported emotional exhaustion, citing workload and inflexible schedules as primary factors (3). The COVID-19 pandemic further disrupted WLB, as remote teaching and increased student supervision blurred work and social boundaries. Similar trends have appeared in Gauteng, where nurse educators experiencing WLB challenges show lower engagement, higher absenteeism, and weaker student mentorship (4). These psychosocial effects extend beyond individual health, directly impacting educational quality and student outcomes.

Gender dynamics also significantly influence WLB experiences. Female nurse educators often encounter increased stress due to societal caregiving expectations, adding to the emotional burden of their work responsibilities (5). Additionally, a lack of organisational support, such as insufficient mental health resources and limited access to employee assistance programmes (EAP), raises anxiety and depression levels among faculty (6, 7).

Despite the known risks of stress and burnout, many NEIs in South Africa lack robust institutional psychosocial support systems. Findings from Limpopo Province highlight institutional neglect as a major stressor, with nurse educators reporting physical health issues, insomnia, and irritability due to heavy workloads (8, 9). Worldwide, strategies such as flexible schedules, mentorship programmes, and mindfulness-based stress reduction (MBSR) have shown promise in improving WLB and lowering burnout (6, 10). However, in resource-limited settings like Gauteng, such initiatives are often given low priority.

A study in Gauteng showed that structured workload redistribution significantly increased job satisfaction and retention among nurse educators (4). While these results are promising, long-term solutions need policy reforms and collaboration between healthcare administrators and nurse educators. Few studies have explored the psychological and social impact of WLB challenges in Gauteng’s vocational training colleges, highlighting the necessity for research and support strategies tailored to the region for nurse educators. This study aims to address the psychosocial impact of WLB that nurse educators in Gauteng encounter and propose strategies to enhance their psychosocial health.

### Theoretical framework

This study explores the psychological impact of WLB among nurse educators within the selected Gauteng NEI, using Spillover Theory (11) as a guiding framework. The theory explains how work-related demands can spill over into personal life, affecting emotional well-being and mental health. In the context of nursing education, negative spillovers occur when academic responsibilities such as teaching, assessment, and administrative responsibilities interfere with social life, leading to stress, burnout, and reduced life satisfaction (12).

Conversely, positive spillover can enhance psychological work-life when professional experiences foster a sense of accomplishment and energy that enriches personal life (13). The study emphasizes the importance of institutional support, including defined work hours, access to mental health services, and mindfulness strategies, in promoting positive spillover and mitigating psychological stress (14, 15). By aligning workplace policies with the principles of Spillover Theory, NEIs can cultivate environments that support both professional effectiveness and personal fulfillment among nurse educators (16). By applying this framework, the study moves beyond merely listing challenges to explaining the underlying process (work-to-life negative spillover) that links organizational conditions to individual psychosocial outcomes. This theoretical positioning directly informs the discussion of findings and the proposed support strategies, which aim to mitigate negative spillover and promote positive work-life integration.

## Research methods and design

This study utilized qualitative, exploratory, and descriptive research design. A qualitative approach was selected as most suitable, given the aim to probe into the complex, subjective lived experiences of nurse educators concerning the psychosocial effects of work-life balance. This method enables a nuanced grasp of meanings, perceptions, and contexts that quantitative approaches alone cannot fully capture (17). An exploratory design was adopted because, although work-life balance is acknowledged as a concern, its specific psychosocial aspects in the context of NEIs in Gauteng, South Africa, remain underexplored. Such a design suits phenomena with limited prior research in each setting, permitting themes to emerge organically from the data.

Complementing this, a descriptive design provided a detailed, systematic portrayal of participants’ characteristics and experiences, faithfully depicting the phenomena as observed (18). This integrated tripartite design was ideally suited to the study’s objective of exploring and describing the psychosocial impact to develop tailored support strategies.

### Research setting

This study was conducted at a government-funded NEI in Gauteng province, South Africa’s economic hub. Despite being the smallest province in terms of geographical size, Gauteng is home to 24.1% of the national population, with approximately 13.4 million residents. The province is divided into two district municipalities and three metropolitan municipalities, including the City of Johannesburg and the City of Tshwane, where the study took place. The NEI, a directorate of the Gauteng Department of Health, operates across six campuses, offering various nursing qualifications that adhere to the National Qualification Framework (NQF) for higher education. The South African Nursing Council (SANC) and the Council on Higher Education (CHE) oversee Education and Training Quality Assurance, with all nursing education regulated by the Nursing Act 33 of 2005. The study focused on two NEI campuses that offer both undergraduate and postgraduate programmes, providing insight into the specific context of nursing education in this setting (17, 19).

### Study population and sampling strategy

The population consisted of nurse educators employed at a specific NEI in Gauteng province. The inclusion criteria specified nurse educators who taught at campuses offering nursing programmes of six months or longer and who were willing to participate. The exclusion criteria were nurse educators involved only in curriculum development, those with less than six months of teaching experience, and those who were unavailable or unwilling to take part. A purposive non-probability sampling method was used to select participants knowledgeable about WLB in nursing education. The final sample included 16 nurse educators, determined by data saturation, which was reached when no new information emerged—after 13 interviews, with three additional interviews conducted to confirm that no new themes were developing (20).

### Data collection

Data were gathered through face-to-face, semi-structured interviews guided by an interview protocol. Probing was used to encourage detailed responses (18) from participants. Before data collection, ethical approval was received from Sefako Makgatho Health Sciences University, and permission was obtained from the Department of Health, the NEI, and its campuses. Participants were recruited during skills development sessions at NEI campuses, informed about the study’s objectives and benefits, and signed informed consent forms, including consent for audio recording. Interviews, conducted in English, lasted 30–45 min in quiet, private settings to ensure confidentiality and reduce distractions. Field notes documented contextual and non-verbal cues. The interview focused on nurse educators’ experiences with WLB and support strategies within the selected NEI (20).

The data collection process encountered several constraints reflecting the pressures faced by nurse educators. Only two of the six targeted campuses participated due to competing priorities, with two campuses inactive and access denied by management at the others, citing educators’ focus on assessments. Scheduling interviews during less busy periods was necessary, as securing uninterrupted time was difficult. The sensitive nature of the topic also led to emotional responses; the trained researcher allowed pauses during interviews and reassured participants of their right to withdraw, often resulting in them feeling lighter after the sessions. These challenges limited the data collection scope, potentially affecting the transferability of findings to other NEIs.

### Data analysis

Thematic analysis followed Braun and Clarke’s (21) six-phase approach, which was well-suited to exploring the psychosocial impact of WLB among nurse educators. The analysis proceeded systematically through each phase:

Phase 1: Familiarization with the data. All interviews were transcribed verbatim by the researcher immediately after each session to preserve participants’ voices and contextual nuances (22). The research team read and re-read transcripts multiple times while listening to audio recordings to ensure accuracy and develop a deep familiarity with the data’s richness.
Phase 2: Generating initial codes. Open coding was conducted systematically, with the researcher identifying meaningful segments of text relevant to the research question. Initial codes were created inductively, capturing both semantic (explicit) and latent (underlying) meanings. This process produced initial codes, documented in a coding manual with definitions and example quotes (36).
Phase 3: Searching for themes. The initial codes were grouped and sorted into potential themes. This involved examining relationships between codes and organizing them into broader thematic categories. Visual mapping using thematic networks helped identify overarching patterns and connections among codes. Preliminary themes were reviewed against coded extracts to ensure coherence.
Phase 4: Reviewing themes. The potential themes were refined through an iterative review at two levels: first, confirming that the extracts within each theme formed a consistent pattern; second, ensuring each theme accurately reflected the dataset. Themes were adjusted, merged, or discarded based on this process. This phase continued until data saturation was reached, with no new themes emerging after the 13th interview; three additional interviews were conducted to verify saturation (23).
Phase 5: Defining and naming themes. Each theme was clearly described, with the researcher explaining its core, scope, and importance. Sub-themes were identified to capture detailed aspects within major themes. This led to two main themes, each with two sub-themes, representing the main challenges and experiences reported by participants.
Phase 6: Producing the report. The final phase involved writing the analytical narrative, selecting compelling verbatim quotes that illustrate each theme while protecting participant anonymity. The analysis combined descriptive accounts with interpretive insights, linking findings back to the research question and theoretical framework.

To ensure analytical rigor, an independent coder with expertise in qualitative research conducted a parallel analysis of randomly selected transcripts without collaboration with the researcher. The independent coder developed separate codebooks and thematic structures. Subsequently, both parties convened for a consensus meeting where codes and interpretations were compared. Disagreements, which primarily concerned the phrasing rather than the substance of themes, were resolved through discussion and reference to the raw data.

### Trustworthiness

Trustworthiness was maintained by addressing five criteria: credibility, transferability, dependability, confirmability, and authenticity (20). Credibility was ensured through prolonged engagement with the field, peer review, data triangulation, and expert supervisory oversight. Transferability was supported by purposive sampling with clear inclusion/exclusion criteria and detailed contextual descriptions to allow application in similar contexts. Dependability was promoted by using a pretested interview guide, consistent procedures, and maintaining a comprehensive audit trail. Confirmability involved independent coding, literature support, and data audits. The researcher and independent coder compared analyses and resolved differences, aligning with recommended validation strategies (24). Authenticity was demonstrated by presenting verbatim participant quotes and ensuring fairness in recruitment and reporting, giving an ethical and balanced account of the participants’ lived experiences.

### Ethical considerations

Ethical clearance was granted by the Sefako Health Sciences University Research Ethics Committee (SMUREC/H/249/2023: PG). Additional permission for data collection was obtained from the National Health Research Database (GP202208010) and the designated NEI. The study adhered to fundamental ethical principles, maintaining respect for human dignity by ensuring that nurse educators participated voluntarily with informed consent after being fully briefed on the study’s purpose and procedures. Scientific integrity was maintained by accurately recording and reporting findings. Beneficence was prioritized by protecting participants from harm or exploitation, minimizing discomfort, and guaranteeing confidentiality. The principle of justice was upheld by safeguarding participant privacy through confidentiality measures and anonymity, utilising coded identifiers to protect personal data throughout the research process.

## Results

### Demographic data

The demographic profile is outlined in Table 1. The 16 participants, mostly women (15 of 16), were primarily aged 50–59, with ages ranging from 36 to 64. Most held bachelor’s degrees (9), followed by master’s (5), PhDs (2), and one honours degree. Their experience ranged from 1.5 to 21 years, representing both early-career and seasoned professionals. This profile reflects a mature, well-qualified, and experienced group supporting the study.

**Table 1:** Summary of demographic data of participants.

Participant	Age range	Gender	Highest qualification	Experience (years)
1	36–40	Female	Master’s (M. N. Ed)	4
2	55–59	Female	Bachelor’s (B. Cur N. Ed)	11
3	45–49	Female	Bachelor’s (B. Cur N. Ed)	8
4	50–54	Female	Bachelor’s (B. Cur N. Ed)	11
5	55–59	Female	PhD	8
6	50–54	Female	Master’s (M. N. Ed)	11
7	55–59	Female	PhD	7
8	55–59	Female	Honours	18
9	55–59	Female	Bachelor’s (B. Cur N. Ed)	9
10	60–64	Female	Bachelor’s (B. Cur N. Ed)	21
11	55–59	Female	Bachelor’s (B. Cur N. Ed)	14
12	60–64	Female	Bachelor’s (B. Cur N. Ed)	16
13	50–54	Female	Master’s (M. N. Ed)	1.5
14	45–49	Female	Bachelor’s (B. Adv Nurs. Sc)	1.5
15	40–44	Female	Master’s (M. N. Ed)	8
16	50–54	Male	Master’s (M. N. Ed)	16

### Themes and sub-themes identified in the interviews

The results in Table 2 show significant psychosocial stress among participants. Nurse educators experienced increased stress and burnout, reporting emotional exhaustion, physical and mental health issues, and growing frustration with their work. Participants also pointed out a lack of emotional and psychosocial support, citing limited access to staff counseling services, insufficient academic support structures, and ongoing social challenges. These stressors adversely affected both their work and social lives.

**Table 2:** Summary of themes and sub-themes.

Themes	Sub-themes
1. Increased stress and burnout	Physical and Mental Health Consequences Emotional exhaustion and frustrations with the work
2. Inadequacy of emotional and psychosocial support	Limited staff counseling services Inadequate study support and social challenges

#### Theme1: increased stress and burnout

The theme highlights significant challenges in NEI, including difficulties in attracting nurse educators to teaching roles due to a perceived lack of value compared to clinical practice. Additionally, they emphasize the importance of maintaining a balance between work and social life, as work-related stress can negatively impact overall health and well-being.

Participant 11: *“It’s depressing for some lecturers. When you encourage people to join nursing education, they often refuse, saying they’d rather stay in clinical practice—because they don’t see the value in what we do.”*

Participant 10: *“We need to balance work and personal life because they’re deeply interconnected. Stress from work affects home life and can lead to high blood pressure and other illnesses commonly linked to workplace stress.”*

##### Subtheme 1.1: physical and mental health consequences

The subtheme revealed the toll of work-related stress and exhaustion on nurse educators’ overall well-being. Participants noted that physical and mental exhaustion impacted productivity. The demanding nature of work affected nurse educators’ ability to function optimally, underscoring the critical need for a WLB. This balance was crucial for maintaining physical and mental well-being. Participants’ experiences illustrated the far-reaching consequences of neglecting this balance.

Participant 2: *“It affects you physically, mentally, and socially—especially your family life. We should stop working once we clock out, go home, and return the next day refreshed.”*

*Participant 10: “You take work home, which impacts your psychosocial well-being because you are married and need to support your husband and children, while work remains very demanding.”*

Participant 14: *“You can become physically and mentally exhausted, which affects your productivity. Someone who’s well-rested can work effortlessly.”*

##### Subtheme 1.2: emotional exhaustion and frustrations with the work

The subtheme revealed that poor WLB among nurse educators contributed to psychological strain. Participants reported extreme fatigue, emotional exhaustion, and reduced motivation, often linked to feeling undervalued and unsupported. These challenges affected personal well-being and led to increased absenteeism.

Participant 12: *“The work nurse educators do is both emotionally draining and intellectually demanding. Carrying that dual burden is incredibly heavy.”*

Participant 4: *“Sometimes I feel so exhausted that I have to plan to take sick leave.”*

Participant 1: *“When it comes to work, support for nurse educators is essential. If I feel undervalued—even something as minor as a sneeze—I’ll stay away from work.”*

#### Theme 2: inadequacy of emotional and psychosocial support

Theme 2 highlighted the urgent need for psychological and emotional support for nurse educators who were facing academic pressures. While management was not viewed as solely responsible, NEI needs to ensure easy access to resources like employee assistance programmes. Creating supportive environments where nurse educators could freely seek help was essential for promoting mental health and maintaining professional effectiveness.

Participant 3: *“We are human beings; we need to be informed about meetings on time. If you don’t prioritize work-life balance, you’re putting your health at risk.”*

Participant 11: *“To say that the manager comes and changes the planning, you ask yourself why, why? What are we doing as nurse educators? Now, this person is oppressing us rather than supporting us.”*

Participant 15: *“As nurse educators, we also need psychological and emotional support. I wouldn’t say management is solely responsible for providing it, but there should be easier ways for employees to access the Employee Assistance Programme.”*

##### Subtheme 2.1: limited staff counseling services

The subtheme revealed that participants faced emotional strain in their roles, felt overwhelmed by the demands of academic life. Participants highlighted the need for accessible emotional support services, noting that psychological distress can affect behavior and performance before it becomes visibly apparent. The study also emphasized the importance of managerial support in helping educators cope with stress and maintain a WLB.

Participant 9: *“I don’t think student counselling should be limited to students. When you’re overwhelmed, you feel it before anyone asks why you’re acting out. That’s when we need to make use of the resources available to us.”*

Participant 10: *“You can end up developing a psychosomatic disorder, which is one of the work-related illnesses we face.”*

Participant 13: *“We are dying of work, ma’am, and hopefully our managers may also support us when we are overwhelmed. Emotional support is needed in achieving work-life balance”.*

##### Subtheme 2.2: inadequate study support and social challenges

Participants highlighted how inadequate study support and social challenges disrupted psychological WLB. The lack of institutional flexibility and pressure to perform despite personal struggles exacerbates stress, undermining well-being and academic productivity. Additionally, participants faced pressure to meet work and academic demands despite personal struggles.

Participant 7: *“Why can’t we get what’s called a sabbatical, like they do in universities? We want to be on the same level as other higher education institutions, but we don’t have access to sabbaticals. Maybe it’s due to budget constraints, but I strongly believe this needs to be investigated.”*

Participant 8: *“Support remains a challenge. For example, I might be dealing with a personal issue, yet I’m still expected to carry on with academic responsibilities. We’re expected to perform at work regardless of our struggles.”*

Participant 16: *“I find personal development challenging because we are required to develop ourselves through both work and further studies. Now, balancing work, studies, and family responsibilities creates a strain. Additionally, there is a lack of support from our managers, which leaves us feeling caught in the middle.”*

## Discussion

This study’s findings align closely with Spillover Theory, particularly illustrating negative work-to-life spillover among nurse educators. Intense role demands and perceived lack of institutional support (Theme 1) act as work stressors that deplete psychological resources, manifesting health issues (e.g., hypertension, exhaustion) and emotional burnout that disrupt WLB. Inadequate psychosocial support (Theme 2)—such as limited counseling or sabbaticals—fails to buffer these effects, allowing stressors to erode personal well-being. The following discussion elaborates these dynamics, connects them to existing literature, and explores targeted interventions as boundary-spanning resources to reduce negative and foster positive spillover, while also addressing the significant concerns raised by participants regarding stress, burnout, and WLB.

Nurse educators highlighted how their roles demand substantial intellectual and emotional labor that often goes unrecognized, creating an overwhelming dual burden exacerbated by insufficient institutional support. This led to reported fatigue, decreased motivation, and withdrawal tendencies when feeling undervalued. These insights align with recent research showing burnout’s prevalence among nurse educators and its negative effects on retention and productivity (25).

The interconnectedness of work and social life was emphasized, with several participants noting that stress from the workplace often led to health complications such as high blood pressure and psychosomatic disorders. This was consistent with global data showing that chronic workplace stress contributed to the development of cardiovascular and mental health conditions (26). The World Health Organization (27) further underscores the economic and social costs of untreated workplace stress, estimating billions in lost productivity annually due to anxiety and depression.

Participants also voiced frustration over the lack of institutional mechanisms to support recovery and rest. The absence of sabbaticals and flexible work arrangements was seen as a barrier to achieving WLB and professional effectiveness. Nurse educators expressed that they felt compelled to continue working despite personal challenges, leading to emotional exhaustion and, in some cases, the need to plan for sick leave simply to recuperate. These findings reflect broader calls for policy reform in academic institutions, where rigid structures often fail to accommodate the human needs of nurse educators (28).

The inadequacy of emotional and psychosocial support was another prominent concern. Participants emphasized the need for accessible psychological services, noting that distress often manifests subtly before it becomes visible. The current support systems, such as Employee Assistance Programmes (EAPs), were described as difficult to access or poorly communicated. While some acknowledged that management may not be solely responsible for providing emotional support, there was a clear consensus that NEIs must do more to ensure these resources are available. This aligns with recommendations from Borcherds and Cloete (29), who advocate for tailored EAPs that address the diverse emotional needs of employees and promote psychological safety.

Moreover, the study revealed that emotional strain is compounded by inadequate study support and social challenges. Participants described feeling overwhelmed by academic responsibilities and unsupported in navigating personal difficulties. The lack of flexibility in institutional policies, such as the absence of sabbaticals, was seen as a significant contributor to stress. These insights resonate with findings from Heunis et al. (30), who emphasize the importance of structured emotional debriefing and mentorship in nursing education to mitigate psychological distress.

Emotional exhaustion, physical health deterioration, and reduced productivity affect nurse educators and compromise the quality of education and institutional stability. The erosion of professional identity and motivation among nurse educators poses a serious threat to the sustainability of academic nursing programmes. As highlighted by the WHO’s Working for Health Action Plan (2022), safeguarding the health workforce requires systemic investment in mental health support, flexible work policies, and recognition of emotional labor.

Nurse educators can benefit from the adoption of flexible work policies, including sabbaticals and protected time for recovery, which have been shown to help reduce burnout and promote well-being (12, 31). Enhancing access to EAPs through clear communication and the creation of supportive WLB environments is crucial to improving mental health outcomes and reducing turnover intentions among nurse educators (32). Furthermore, prioritising psychological safety within academic settings allows nurse educators to express concerns freely without fear of judgment, which contributes to better WLB and less occupational stress (33, 34). The incorporation of mental health training, mentorship, and structured support systems is essential to fostering resilience and professional development among nurse educators (35). Importantly, a cultural shift is necessary to acknowledge and value the emotional and intellectual contributions of nurse educators, encouraging their engagement and supporting long-term retention through organisational support and compassionate leadership practices (12).

## Conclusion

The study concludes that poor work-life balance has a profound psychosocial impact on nurse educators, leading to emotional fatigue, lower job satisfaction, and compromised mental health. The absence of sufficient institutional support worsens these issues. It is recommended that Nursing Education Institutions implement strategies, including accessible counseling services, structured academic support, and initiatives promoting work-life balance, to improve the psychosocial well-being of nurse educators.

## Data Availability

The original contributions presented in the study are included in the article/supplementary material, further inquiries can be directed to the corresponding author.
